# Supplementary material for: Effects of Dupilumab on Itch-Related Events in Atopic Dermatitis: Implications for Assessing Treatment Efficacy in Clinical Practice
Source: Cells. 2023 Jan 5;12(2):239. doi: 10.3390/cells12020239 (PMC9857157; doi:10.3390/cells12020239)
Supplement: Supplementary file 1 [file cells-12-00239-s001.zip › cells-2059733-supplementary.pdf]

cells

MDPI

Article

Effects of Dupilumab on Itch-Related Events in Atopic Dermatitis: Implications for Assessing Treatment Efficacy in Clinical Practice

Ryoma Kishi [1,2], Sumika Toyama [1], Mitsutoshi Tominaga [1,3], Yayoi Kamata [1,3], Eriko Komiya [1], Takahide Kaneko [2], Yasushi Suga [2,3] and Kenji Takamori [1,2,3,*]

[1] Juntendo Itch Research Center (JIRC), Institute for Environmental and Gender-Specific Medicine, Juntendo University Graduate School of Medicine, 2-1-1 Tomioka, Urayasu, Chiba 279-0021, Japan

[2] Department of Dermatology, Juntendo University Urayasu Hospital, 2-1-1 Tomioka, Urayasu, Chiba 279-0021, Japan

[3] Anti-Aging Skin Research Laboratory, Juntendo University Graduate School of Medicine, 2-1-1 Tomioka, Urayasu, Chiba 279-0021, Japan

* Correspondence: ktakamor@juntendo.ac.jp; Tel.: +81-47-353-3171

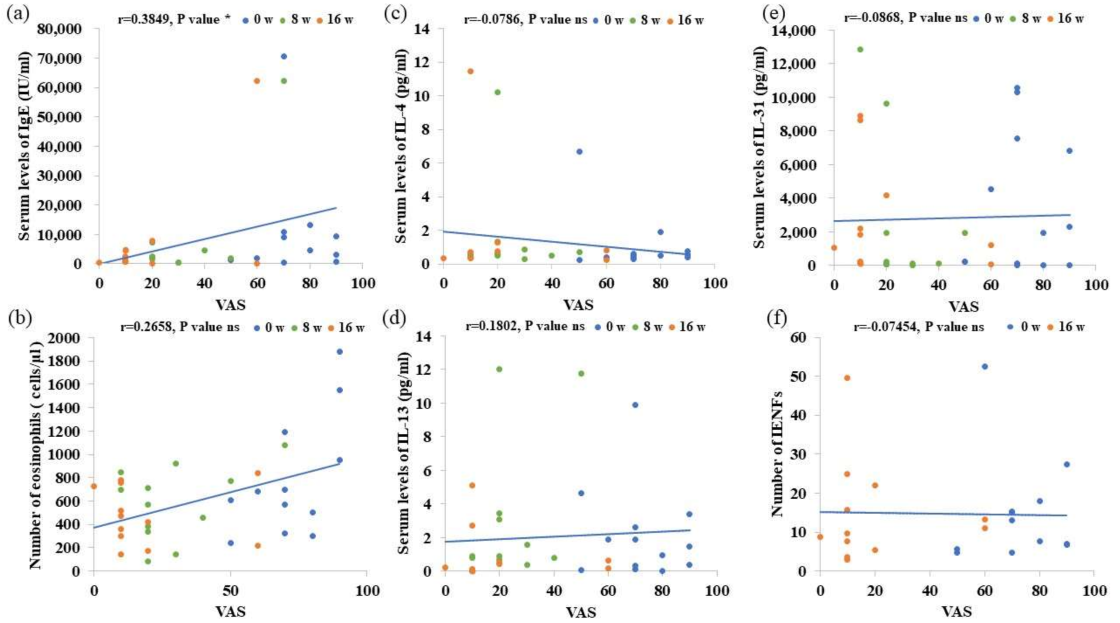

Supplementary Figure S1. Correlation analysis of VAS scores with IgE, eosinophils, IL-4, IL-13, IL-31 and IENFs. (**a**-**f**) Correlation analysis of VAS scores with (**a**) serum IgE levels, (**b**) Number of eo-sinophils in peripheral blood, (**c**) serum levels of IL-4, (**d**) serum levels of IL-13, (**e**) se-rum levels of IL-31, and (**f**) number of IENFs. Data are shown as the mean ± standard error of the mean (n=12 per group). Coefficients (r) were assessed by Spearman's rank correlation test. *P<0.05. VAS, visual an-alogue scale; EASI, eczema area and severity index; IgE, immunoglobulin E; IL, interleukin; IENFs, intraepidermal nerve fibers; TARC, thymus and activation-regulated chemokine; ns, not significant.
